# Coblation-Assisted Endoscopic Endonasal Approach for Restenosis in Unilateral Choanal Atresia: A Case Report

**DOI:** 10.7759/cureus.96414

**Published:** 2025-11-09

**Authors:** Methodios T Stavridopoulos, Vaios T Kouliopoulos, Vasileios Chalkiadakis

**Affiliations:** 1 Otolaryngology - Head and Neck Surgery, 417 Army Equity Fund Hospital, Athens, GRC

**Keywords:** coblation, endonasal approach, restenosis, revision surgery, unilateral choanal atresia

## Abstract

Choanal atresia (CA) is a rare congenital disease caused by the developmental failure of the posterior nasal cavity to communicate with the nasopharynx. The unilateral type, affecting mainly the right nasal cavity, can remain undiagnosed for years. To date, no gold standard surgical approach has been established, and the risk of requiring revision surgery remains considerable. We present the case of a 33-year-old male patient who complained of chronic right-sided nasal blockage and discharge, which initiated a few years after pediatric surgery for ipsilateral CA. Rigid nasal endoscopy revealed his symptoms were caused by restenosis. The patient underwent successful transnasal endoscopic coblation-assisted surgical treatment without using stents. There were no postoperative complications reported, and one year into follow-up, no restenosis is evident. This case demonstrates the safety and efficacy of the coblation-assisted endonasal approach.

## Introduction

Choanal atresia (CA) constitutes a rare congenital malformation caused by complete obstruction of the posterior choana and occurs with an incidence of 1:5000-8000 live births [1]. There is a female-to-male predominance (ratio 2:1), and in most cases, the malformation is of mixed bony and membranous tissue (70%), while in the remaining cases, it is of pure bony tissue, with purely membranous tissue atresia considered extremely rare [2,3]. Bilateral CA, which accounts for 30-40% of cases, is considered a life-threatening emergency in infants [4]. On the contrary, the unilateral type, which is more common and usually located in the right nasal cavity (71%), may be diagnosed later in life [5]. Computed tomography (CT) is essential in both types to facilitate the diagnosis, and surgical management is needed to restore choanal patency [4]. Currently, there is no consensus on a definitive surgical approach for CA [6]. Numerous case reports and case series, as well as a few review articles, exist in the literature, describing different techniques and reporting various rates of restenosis that greatly fluctuate between 12% and 54% [7]. Similarly, a single universally accepted revision protocol has not been adopted, despite a recent shift in stentless endoscopic transnasal approaches that take advantage of mucosal flaps [8]. In recent years, coblation, a bipolar radiofrequency-based technique operating at comparatively low temperatures through molecular bond disruption and tissue dissolution, has gained application across various rhinologic procedures - including cases of post-radiation choanal stenosis - due to its advantages in enhancing intraoperative visualization and reducing postoperative pain [9-11].

## Case presentation

**Figure 1:**
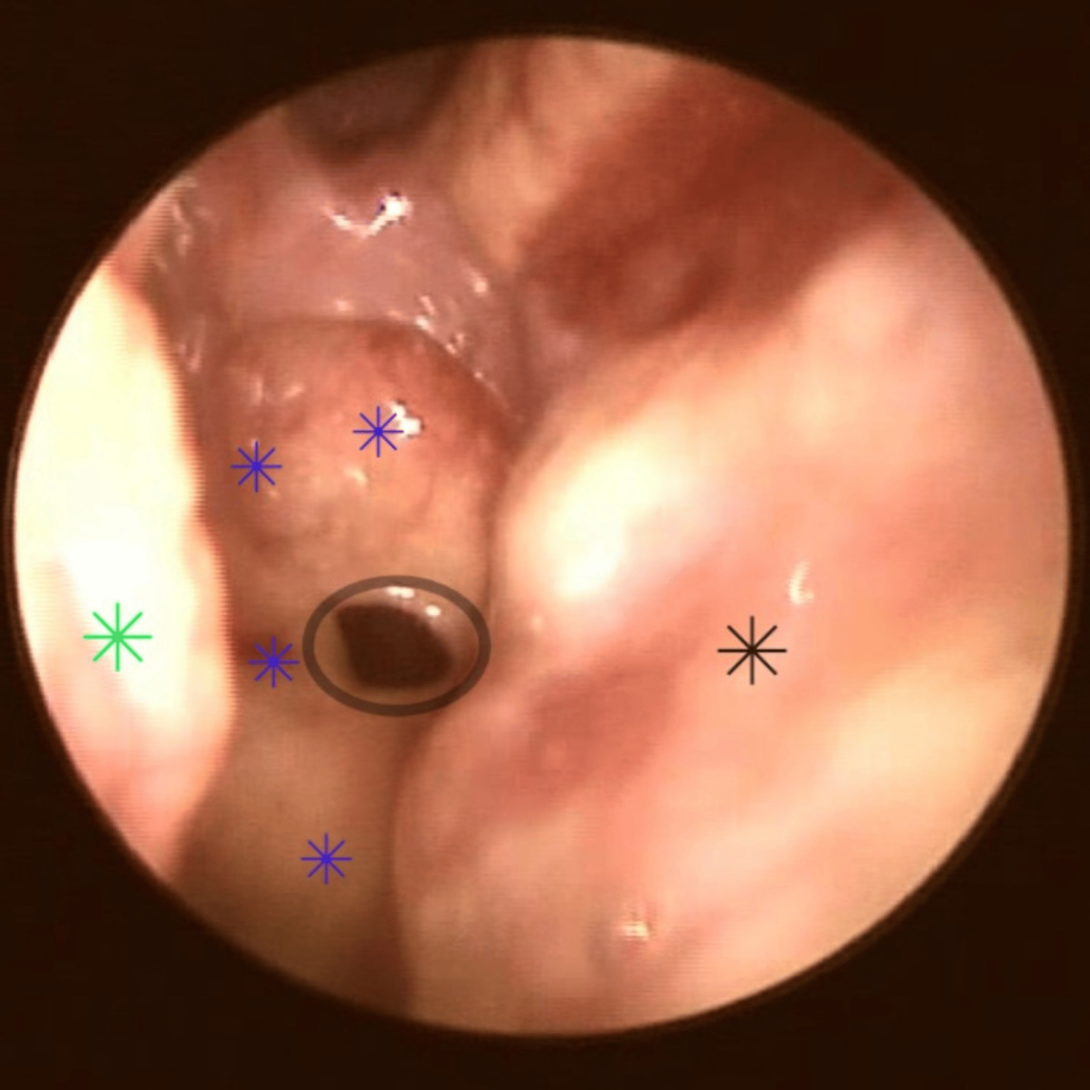
Rigid nasal endoscopy of the right side Near atretic choanal plate (blue stars), narrow orifice of restenosis connecting the nostril with the nasopharynx (black eclipse), nasal septum (black star), inferior turbinate (green star).

**Figure 2:**
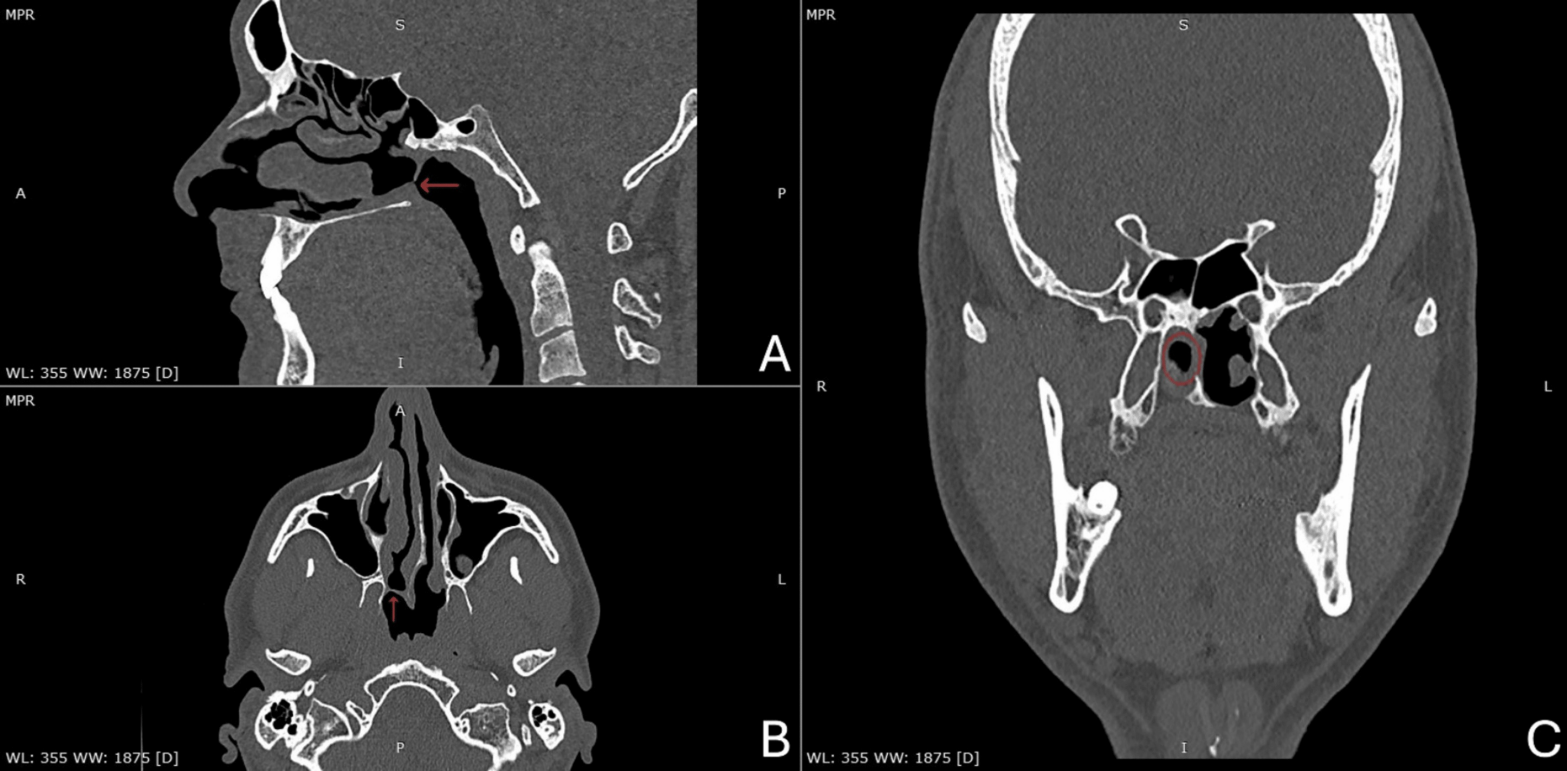
Multiplanar view of sinus CT scan A: Sagittal view. Near-complete obstruction of the right posterior choana from membranous tissue (red arrow). B: Axial view. Near-complete obstruction of the right posterior choana from membranous tissue (red arrow). C: Coronal view. Near-complete obstruction of the right posterior choana from membranous tissue (red eclipse).

A 33-year-old male patient presented to the ENT outpatient clinic with complaints of chronic right-sided nasal congestion and watery discharge. The patient had undergone surgery at four years of age due to unilateral CA of the right side. Unfortunately, medical records from the pediatric hospital could not be retrieved. Rigid nasal endoscopy revealed restenosis of the right side posterior choana with a very narrow opening (Figure 1). CT depicted a unilateral posterior nasal obstruction of membranous tissue (Figure 2). The patient underwent successful surgical treatment utilizing a coblation-assisted endoscopic approach.

**Figure 3:**
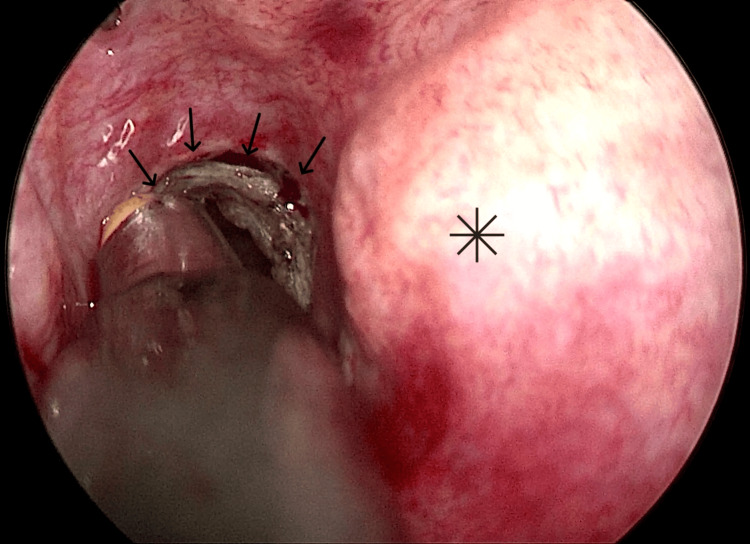
Coblation-assisted removal of choanal restenosis Removal of granulation tissue in the choana (black arrows), nasal septum (black star).

Under general anesthesia and endoscopic guidance of a 0º, 4 mm telescope, the inferior turbinate of the right side was ablated using a radiofrequency probe and was fractured outward and inward using the Freer elevator to achieve maximum visualization of the choana. Afterwards, submucosal injections of lidocaine 1% with adrenaline 1:100.000 were performed on the posterior septum. Using the coblation probe, the granulation tissue that blocked the posterior choana was removed and simultaneously cauterized, providing minimal intraoperative bleeding (Figure 3). The resection’s operating limits were defined inferiorly by the nasal floor, superiorly by the vaginal process of the medial pterygoid plate of the sphenoid bone, laterally by the medial pterygoid plate of the sphenoid bone, and medially by the posterior septum.

**Figure 4:**
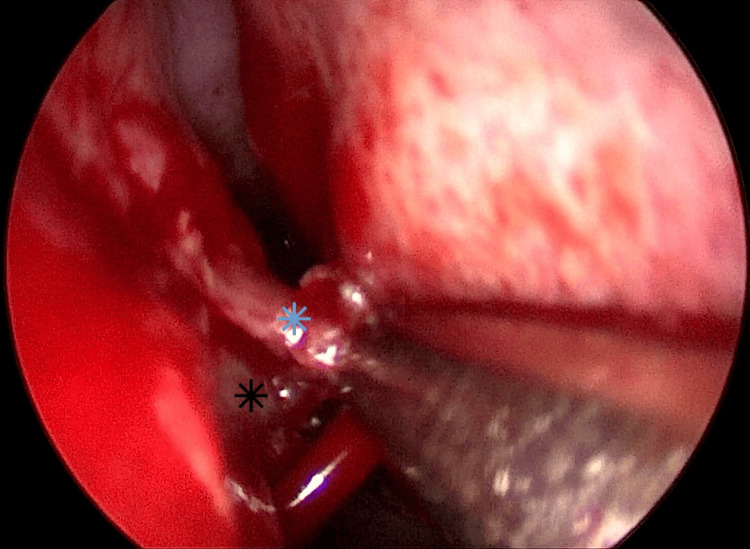
“Π”-shaped incision of the mucoperiosteum of the posterior septum on the left side Truncated septal flap from the posterior septal branch of the sphenopalatine artery (blue star), posterior bony septum (black star).

**Figure 5:**
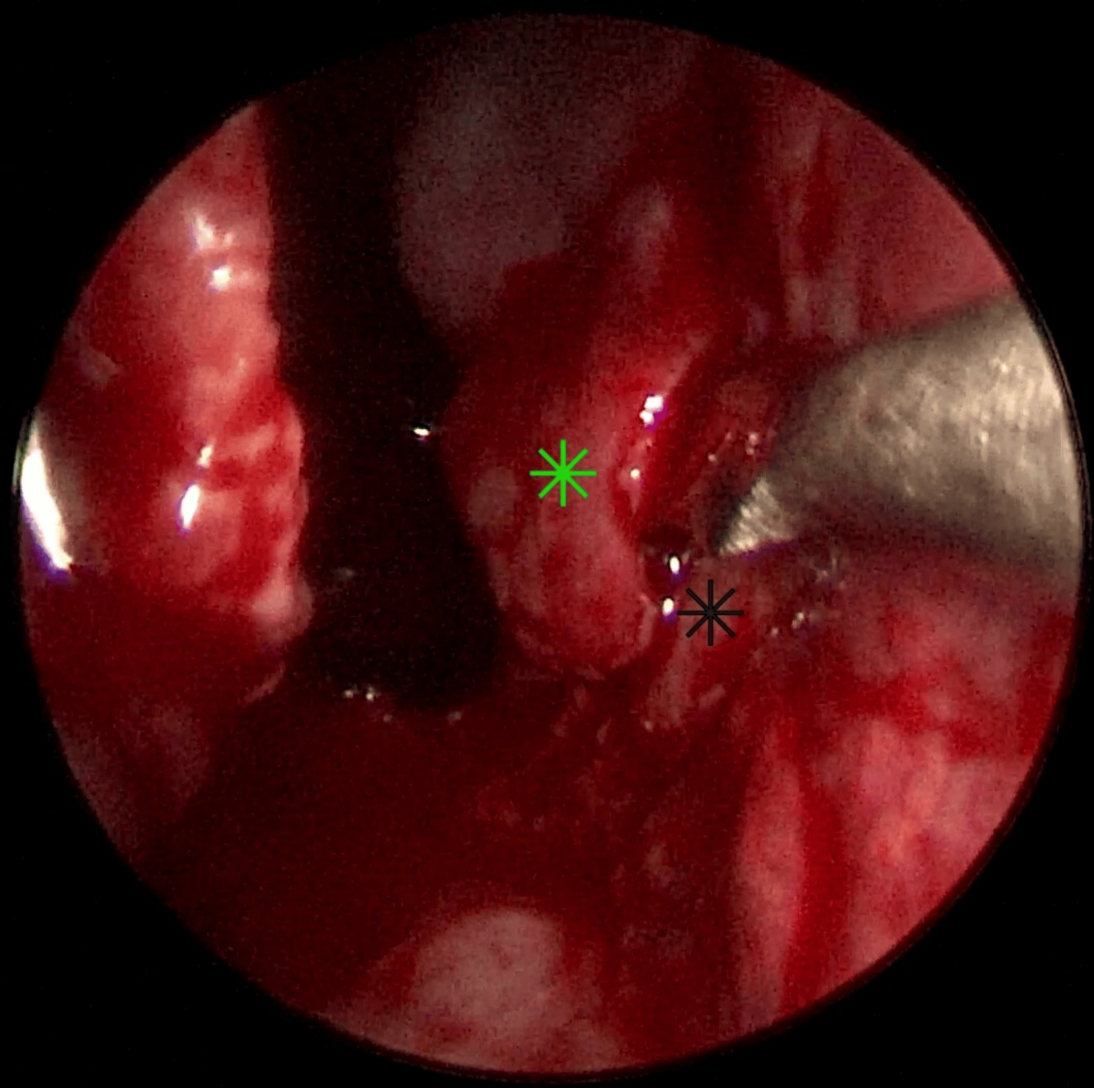
“U”-shaped incision of the mucoperiosteum of the posterior septum on the right side Randomly based flap that retained its vascular supply from the superior septum (green star), posterior bony septum (black arrow).

**Figure 6:**
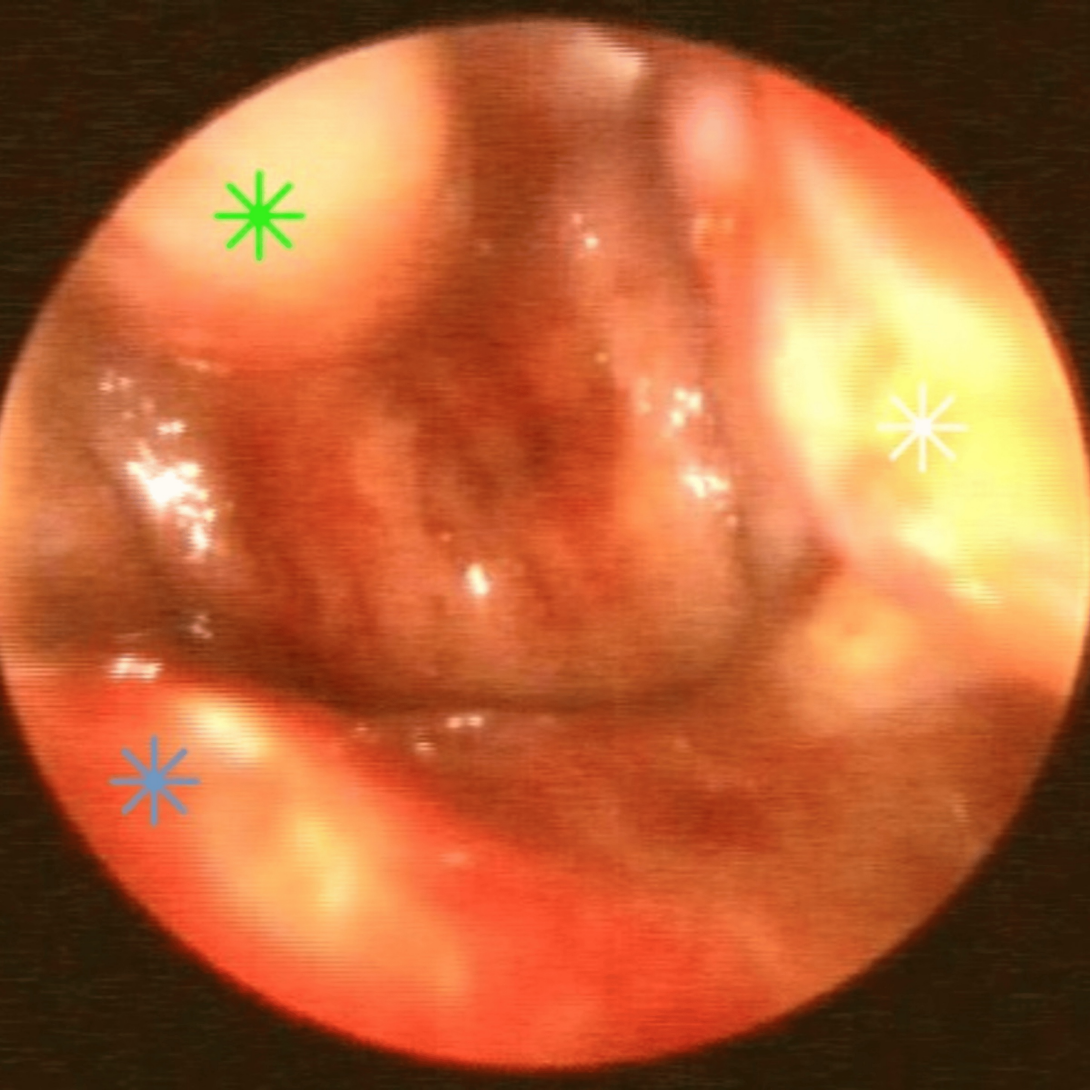
Utilization of the cross-over flap technique Covering of the superior border of the remnant bony septum with the randomly based flap (green star), covering of the inferior border with the truncated flap (blue star), left inferior turbinate (white star).

Afterwards, to ensure the patency of the re-opened choana, the posterior part of the septum was also removed, utilizing the cross-over flap technique. Using the microsurgical scalpel, a “Π”-shaped incision was made through the mucoperiosteum of the posterior septum on the left side, and the mucoperiosteum was elevated using the Freer and Cottle elevators. This created a truncated septal flap from the posterior septal branch of the sphenopalatine artery (Figure 4). A similar “U”-shaped incision was made ~1 cm anteriorly of the near-atretic plate on the right side, creating a randomly based flap that retained its vascular supply from the superior septum and allowing visualization of the bony portion of the posterior septum (Figure 5). Utilizing the back-biting forceps and the Kerrison punch, the posterior part of the vomer was removed. The randomly based flap was then used to cover the superior border of the remnant bony septum, and the truncated flap provided cover for the inferior border (Figure 6). The flaps were secured in place using fibrin glue and hemostatic bioabsorbable gauze. There were no packing, stent, topical mitomycin C, or corticosteroids applied.

**Figure 7:**
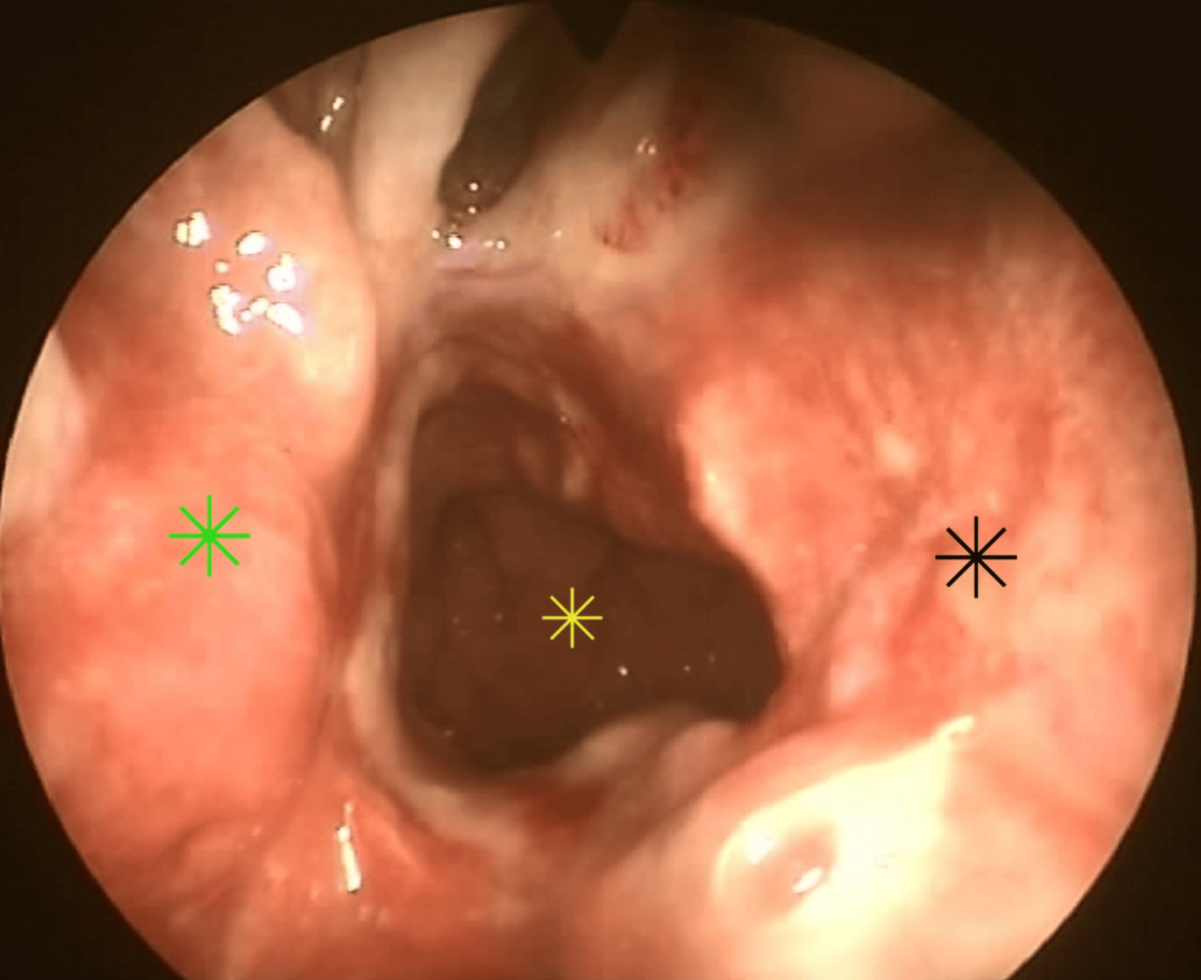
One-month postoperative endoscopic view with no restenosis evident Nasopharynx (yellow star), right inferior turbinate (green star), nasal septum (black star).

**Figure 8:**
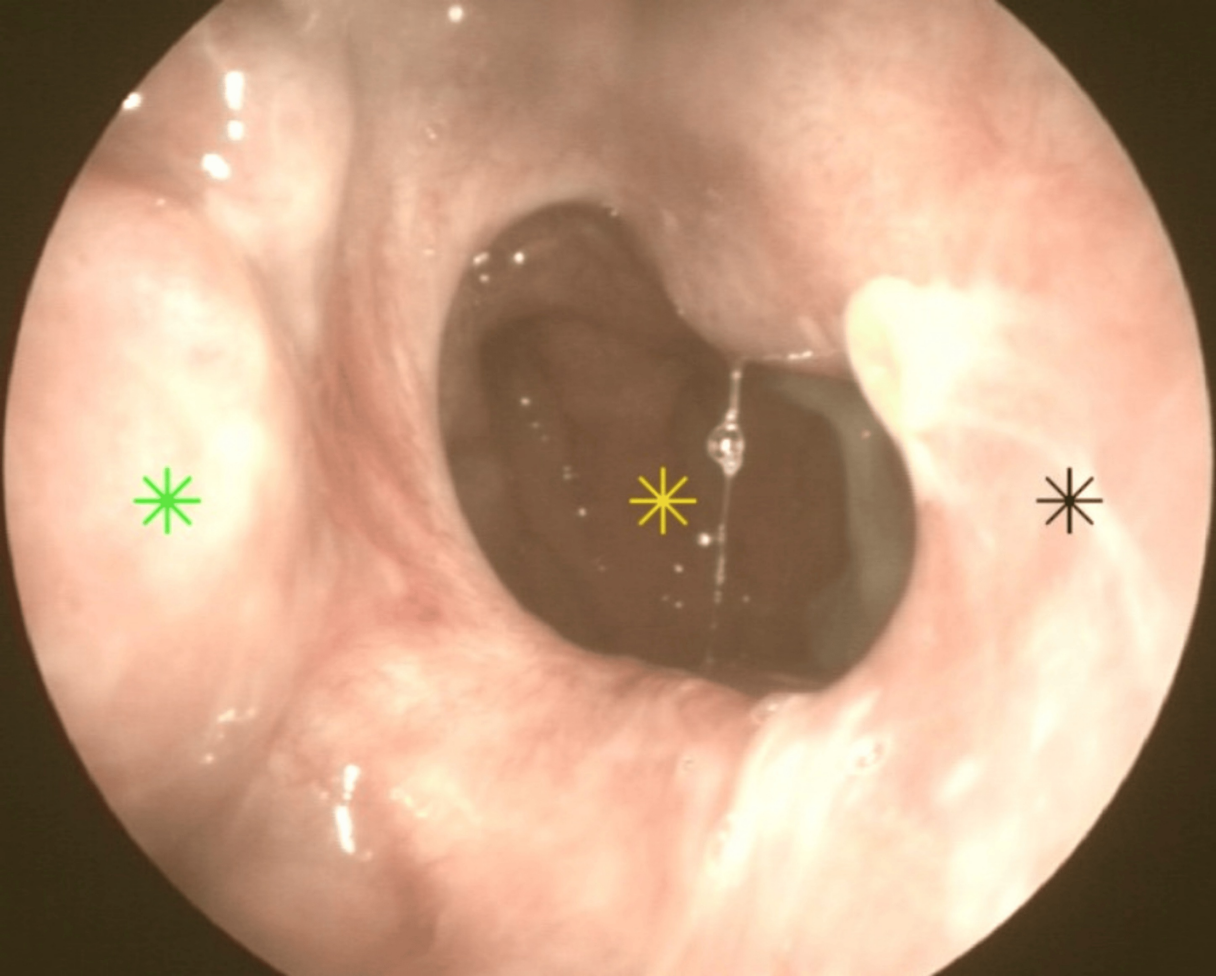
One-year postoperative endoscopic view with no restenosis evident Nasopharynx (yellow star), right inferior turbinate (green star), nasal septum (black star).

The patient reported no postoperative complications and was discharged the following day. He was advised to perform nasal irrigations with a saline solution at least four times daily for the first month after surgery. Close follow-up was maintained, weekly during the first month and monthly afterwards, to monitor healing progress (Figure 7). To date, one year post-op, there is no evident restenosis of the choana (Figure 8). The patient highlights a significant improvement in nasal airflow and quality of life.

## Discussion

Primary surgical management of CA includes various procedures, and their success can be measured based on multiple parameters, the most important being the avoidance of restenosis [4]. To this day, no consensus has been reached regarding the optimal surgical approach [2]. Both transoral and transnasal approaches have been described in the literature with or without the use of stents (manufactured from various materials), topical steroids, and mitomycin C [5].

The trans-palatal and trans-antral approaches offer adequate exposure and field for operation, but are not considered optimal nowadays, due to high risk of complications such as bleeding, fistulas, growth deformities of the maxilla, palatal bone, and upper teeth [2,8]. Transnasal and transeptal approaches are the most commonly used currently, taking advantage of endoscopes, instruments of cold steel, cauterizing tools, diamond drills, CO_2_ lasers, and dilators [2,6,8]. Various techniques have been described depending on the patient’s age, including perforation of the atretic plate followed by dilatation using Hegar or balloon dilators, choanoplasty with microdebridement and/or drilling of the atretic choana, and resection of the posterior portion of the vomer, with incorporation of mucosal flaps, stents, or mitomycin C [5].

Implementing stents to retain the fissure created in the place of the atretic choana after surgery has been widely debated in the literature [4]. Some studies argue that without stents, there is a higher possibility of restenosis, while others link the use of stents with postoperative complications such as discomfort, localized infection, ulceration, circumferential scarring, and the formation of granulation tissue, which ultimately lead to restenosis [2,4-6,8,12]. Furthermore, some authors have suggested that other factors, such as the material of the stent, the duration of stenting, the patient’s age, regulation of gastroesophageal reflux, bilateral versus unilateral atresia, and associated syndromes, could affect the emergence of these complications and the chances of successful treatment [2,4,5,8,12]. However, the latest reviews highlight that despite the lack of significant evidence in favor of a specific surgical technique with or without stenting, it is clear that stentless techniques show fewer postoperative complications [4,13]. Unfortunately, in our case, we were not able to determine whether stents were applied during the initial surgery.

Restenosis constitutes a complication influenced by many factors besides the surgical technique employed. Overall, restenosis rates range from 20% to 50%, often necessitating revision surgery [1,14-16]. However, the incorporation of mucosal flaps and stentless approaches has demonstrated improved outcomes, with restenosis rates reduced to 10-30% in some series [2].

Revision surgery remains a clinical challenge, with surgical approaches largely mirroring those used in primary repair. The most commonly employed approach is the transnasal endoscopic one, utilizing a combination of microdebriders, powered drills, and mucosal flap techniques to re-establish a patent choana while minimizing granulation tissue formation [17]. Adjunctive measures such as topical mitomycin C, balloon dilation, and, most recently, bioabsorbable steroid-eluting stents have also been enlisted to reduce restenosis rates [2]. Reported success rates for revision surgeries vary widely, ranging from 65% to 85% [17].

To our knowledge, this report constitutes the first documented case of coblation-assisted endoscopic management of restenosis in unilateral CA. Utilization of coblation has only been reported in primary surgery for bilateral CA once [18] as well as in choanal stenosis following radiation in nasopharyngeal carcinoma [10,11]. The term "coblation" is derived from controlled ablation, emphasizing the precision and controlled nature of tissue removal provided. Coblation uses radiofrequency energy that is passed through a conductive medium, typically saline solution. The saline solution is then “excited,” creating a plasma field composed of highly energized ions. This plasma field manages to break down molecular bonds within soft tissue, thus dissolving it. The main advantage of coblation is the minimization of damage to surrounding healthy tissue, due to its operation at relatively low temperatures of 60°C to 70°C, especially when compared to electrocautery, which may reach temperatures between 400°C and 600°C [9]. Lastly, coblation offers multitasking with one hand, as it provides simultaneously ablation, coagulation, and irrigation, which is extremely helpful in endoscopic surgery [19].

The authors believe that the precise removal of only scar tissue without damaging surrounding structures, as well as the minimal thermal damage that coblation-assisted surgery offered, contributed to the avoidance of further granulation postoperatively. Moreover, the cross-over flap technique was incorporated to remove part of the posterior septum and widen the nostrils' communication to the nasopharynx. Our patient has been evaluated for one year, and the patency of the choana remains the same with no restenosis. Thus, utilization of coblation could prove ideal in CA restenosis cases or in the rare cases of purely membranous tissue CA.

This report is limited by its nature as a single case study and the relatively short follow-up period of one year. Further research involving larger patient cohorts and longer-term outcomes is needed to fully establish the efficacy and safety of the coblation-assisted technique in the management of CA restenosis.

## Conclusions

To our knowledge, this case presents the first ever utilization of the endoscopic endonasal coblation-assisted technique in a revision case of unilateral CA. Our patient experienced significant improvement following surgery with no post-operative complications or signs of restenosis during a one-year follow-up. The approach's key advantages include minimal scarring that does not add to the existing granulation and the avoidance of stent-related complications, such as discomfort, infection, ulceration, and fibrosis. This surgical technique could potentially be a valuable addition to surgical options for restenosis in unilateral CA, particularly in adult patients, as our case highlights its safety and efficacy. Long-term follow-up and further studies comparing this technique to other surgical methods are needed to strengthen its position in clinical practice.
